# *Metarhizium robertsii* Produces an Extracellular Invertase (MrINV) That Plays a Pivotal Role in Rhizospheric Interactions and Root Colonization

**DOI:** 10.1371/journal.pone.0078118

**Published:** 2013-10-21

**Authors:** Xinggang Liao, Weiguo Fang, Liangcai Lin, Hsiao-Ling Lu, Raymond J. St. Leger

**Affiliations:** 1 Department of Entomology, University of Maryland, College Park, Maryland, United States of America; 2 Institute of Microbiology, College of Life Sciences, Zhejiang University, Hangzhou, Zhejiang, China; 3 Tianjin Institute of Industrial Biotechnology, Chinese Academy of Sciences, Tianjin, China; Universidade de Sao Paulo, Brazil

## Abstract

As well as killing pest insects, the rhizosphere competent insect-pathogenic fungus *Metarhizium robertsii* also boosts plant growth by providing nitrogenous nutrients and increasing resistance to plant pathogens. Plant roots secrete abundant nutrients but little is known about their utilization by *Metarhizium* spp. and the mechanistic basis of *Metarhizium*-plant associations. We report here that *M. robertsii* produces an extracellular invertase (*MrInv*) on plant roots. Deletion of *MrInv* (*⊿MrInv*) reduced *M. robertsii* growth on sucrose and rhizospheric exudates but increased colonization of *Panicum virgatum* and *Arabidopsis thaliana* roots. This could be accounted for by a reduction in carbon catabolite repression in *⊿MrInv* increasing production of plant cell wall-degrading depolymerases. A non-rhizosphere competent scarab beetle specialist *Metarhizium majus* lacks invertase which suggests that rhizospheric competence may be related to the sugar metabolism of different *Metarhizium* species.

## Introduction

The entomopathogenic fungus *Metarhizium robertsii* (formerly known as *Metarhizium anisopliae* var *anisopliae* [1]) is currently applied as a biological control agent against various insect pests [2-4]. Recent investigations have revealed that species of *Metarhizium* which are widely distributed in soil also play other ecological roles, including establishing mutualistic interactions with plants as rhizospheric associates [5]. Seed treatment with these *Metarhizium* spp. increases crop yields by killing soil insects [6], inhibiting plant pathogens [7], enhancing the uptake of micronutrients [8], and translocating nitrogen from killed insects to plants [9]. However, *Metarhizium* has yet to be exploited in agriculture as a plant growth enhancer and the mechanistic basis for its many interactions are poorly understood. The mycoparasite *Trichoderma* is the best studied plant root colonizer and the most widely applied fungal plant growth promoter [10,11]. Much effort and expense has been directed at finding better ways to apply both *Metarhizium* spp. (as insecticides) and *Trichoderma* spp. (as plant symbionts). However, as with *Metarhizium*, exploitation of *Trichoderma* has been handicapped by inconsistent field performance [12,13]. More consistent biopesticide products might be achieved based on combinations of microbes that economically achieve breadth of action. To this end, identifying commonalities and differences between *Metarhizium* and *Trichoderma* in their mode of action could be used to facilitate synergistic effects when applied together as a seed treatment. Comparative genomics reveals *Metarhizium* and *Trichoderma* are related [14], but studies to date have shown mechanistic differences in root colonization that suggest they have independently evolved rhizosphere competence. For instance, *Trichoderma* uses a hydrophobin to adhere to roots [15], whereas *Metarhizium* uses an adhesin [16].

Nevertheless, given their shared habitat there should also be similarities and some of these could potentially lead to competition for resources. In soils, both *Metarhizium* and *Trichoderma* exploit various products of plant photosynthesis. The ability to respond to and utilize these products will likely determine their success as plant root colonizers. Sucrose is one of the main carbohydrate products of photosynthesis and has been detected in high concentrations near root tips [17-19]. Several fungi such as *Thermomyces lanuginosus*, *Uromyces fabae*, *Aspergillus niger* and *Trichoderma virens* produce invertases capable of hydrolyzing sucrose to monosaccharides [20-23]. In *U. fabae*, invertase is highly expressed in haustoria during the process of infection in the leaf. Furthermore, plant-derived sucrose is a key component in symbiotic associations between *T. virens* and maize [22].

In the present study, a gene (*MrInv*) with homology to the intracellular invertase of *T. virens* was identified and characterized in *M. robertsii*. *MrInv* encodes a single copy extracellular invertase that is principally responsible for sucrose hydrolysis by *M. robertsii*. Disrupting the gene resulted in poor fungal growth on sucrose, root exudates and rhizospheric soils. However, root colonization by the mutant was increased. We present evidence that this resulted from increased expression of cell wall-degrading enzymes because of reduced carbon catabolite repression in the mutant. Our data suggest that while *MrInv* plays an important role in providing the fungus with a carbon source, this limits the extent of root colonization during *Metarhizium*-plant associations.

## Materials and Methods

### Living Materials and Culture Conditions

*Metarhizium robertsii* ARSEF 2575 and *M. majus* ARSEF 297 wild-type strains (USDA/ARS Collection) were grown and maintained on Potato Dextrose Agar (PDA) (Fluka, USA) at 27 °C. *Escherichia coli* DH5α and *Agrobacterium tumefaciens* AGL-1 were used for DNA cloning and fungal transformation. *Arabidopsis thaliana* eco-type Col-0 seeds were purchased from LEHLE SEEDS (Round Rock, Texas, USA). *Panicum virgatum* (switchgrass) seeds were obtained from OSC seeds (Waterloo, Ontario, Canada). *A. thaliana* and switchgrass seeds were surface-disinfected according to Sauer and Burroughs [24] and Miché and Balandreau [25], respectively. The sterilized seeds were kept at 4 °C overnight to allow for synchronization of growth before fungal inoculation.

### Gene Disruption

A *MrInv* disruption vector pPK2BargfpDMrInv was constructed to knock out the *MrInv* gene in *M. robertsii*. The 5′-end and 3′-end of *MrInv*, cloned by PCR, were inserted into a modified master Ti vector pFBarGFP using the *Xba* I and *Bgl* II/*Eco*R V sites, respectively. The disruption mutant (*⊿MrInv*) was obtained by *A. tumefaciens*-mediated transformation [26]. To complement *⊿MrInv*, a ~ 3.9 kb genomic fragment containing the *MrInv* open reading frame and its flanking sequence was cloned and inserted into the *Eco*R V site of pPK2SurGFP with the *Sur* selective marker under control of a constitutive glyceraldehyde-3-phosphate dehydrogenase (Pgpd) promoter [27,28]. The ⊿*MrInv* revertant (⊿*MrInv-rv*) was obtained by transforming the construct into *⊿MrInv*.

### qPCR Analysis

To quantify gene expression in response to sugars, wild-type *M. robertsii* and *M. robertsii ⊿MrInv* were grown in Sabouraud Dextrose Broth (SDB, Difco) for 30 h, and mycelia were transferred to minimal medium (M100 medium minus glucose) plus 1% sucrose (sucrose cultures). To observe the time course of *MrInv* expression, RNA was harvested from mycelia for qPCR analysis as described previously [29]. The primers used in this study are listed in Table S1.

### Sugar Utilization Assay

The ability of *⊿MrInv* and wild-type strains to utilize different sugars was analyzed according to a modification of the method of Fang and St. Leger [30]. Sterile distilled water plus or minus a nitrogen source (0.1% NaNO_3_) was combined with a carbohydrate (0.1%). The soybean root exudate was prepared as described by Pava-Ripoll et al. [31]. To determine growth rates, mycelial inoculums (0.2 g wet mycelia, approximately 25 mg dry biomass) from SDB cultures were transferred into liquid minimal medium supplemented with 1% sucrose and/or 1% glucose. Mycelia harvested after 12 h growth was used for dry weight determinations.

### Rhizosphere Competence and Root Colonization Assays

Sterile synchronized *P. virgatum* (switchgrass) seeds were inoculated by immersion for 1 h in 1×10^8^/mL^-1^ *M. robertsii* wild-type or *⊿MrInv* spores as described by Wyrebek et al. [32]. The seeds were planted in pots (0.5 g seeds per pot) filled with sterile soil (Scotts Turf Builder Seeding Soil, Scotts Company, USA). Each treatment was replicated three times. To determine fungal survival in bulk soil with or without switchgrass seeds, spore suspensions were spread evenly through soil producing approximately 5 × 10^3^ spores/g^-1^ soil. Half the pots were planted with uninoculated seeds. All the pots were kept in a growth chamber at 25 °C with 14:10 h light:dark cycle, and within two weeks the grass had produced a lawn covering the soil surface. Sterile water was added regularly to avoid drying. The soil population of wild-type and *⊿MrInv* strains was monitored at set intervals by a slight modification of Fang and St. Leger [30]. Briefly, 0.5 g of soil from each pot was collected using a cork borer. This soil contained a high density of roots so the fungus would be existing in overlapping rhizospheres. Soil suspensions were prepared by adding 5 mL 0.05% Tween 80 solution and vortexing vigorously. Aliquots (100 μL) were spread on Rose-bengal selective agar plates and CFUs were determined after 10 days at 27 °C [5].

To assay root colonization, 10-d-old *A. thaliana* seedlings were inoculated in liquid Murashige and Skoog medium (Sigma, USA) containing 5 × 10^6^ spores mL^-1^ *M. robertsii* suspensions. After 48 h incubation, *A. thaliana* roots were collected, and washed three times in 0.05% Tween 80 solution. To observe fungal colonization, individual *A. thaliana* root were stained with fungi lactophenol cotton blue (ENG Scientific, Inc., USA) and mounted on a slide for microscopy. *M. robertsii* root colonization was quantified by a modification of Viterbo et al. [33]. To quantify wild-type and mutant growth, three replicates each containing nine *A. thaliana* roots were weighed and homogenized by vortexing vigorously for 2 min in 500 μL 0.05% Tween 80 solution. Serial dilutions were assayed for CFU on Rose-bengal selective agar plates [5]. The same procedure was applied on switchgrass except that each replicate contained three switchgrass roots from each pot. Roots were weighed and ground with mortar and pestle in 2 mL 0.05% Tween 80 solution. The CFUs were quantified as described above.

### Enzymatic Assays

Enzymatic activities were assayed in filtrates from cultures grown with sucrose or *A. thaliana* seedlings as described previously. The Pr1 subtilisin protease activity was assayed with N-succinyl-Ala-Ala-Pro-Phe-p-nitroanilide [34]. One unit of protease activity was defined as the amount of enzyme that produces 1 μM of para-nitro aniline per minute. Invertase and endochitinase activities were assayed using the EnzyChrom^TM^ Invertase Assay Kit (BioAssay Systems, USA) and the Chitinase Assay Kit, Fluorimetric (Sigma, USA). Total protein content was measured with a Protein Assay Kit II (Bio-Rad, USA). Pectinase activity versus polygalacturonic acid was assayed according to the protocol from Worthington Biochemical Corporation (New Jersey, USA). One unit of pectinase was defined as the amount of enzyme that liberates 1 µmole of D-galacturonic acid from polygalacturonic acid per minute.

### Plant Growth

To assess the effect of *M. robertsii* on switchgrass, stem lengths were measured and leaf chlorophyll content was read using a SPAD-502 Plus chlorophyll meter (Konica Minolta Sensing, Inc. Japan). The plants were harvested after three months, and rhizospheric soils washed off the roots, and root lengths measured as described previously [5]. The dry weight of whole plants was determined as a measure of biomass.

## Results

### Identification and Characterization of an Invertase MrINV in M. *robertsii*

**Figure 1:**
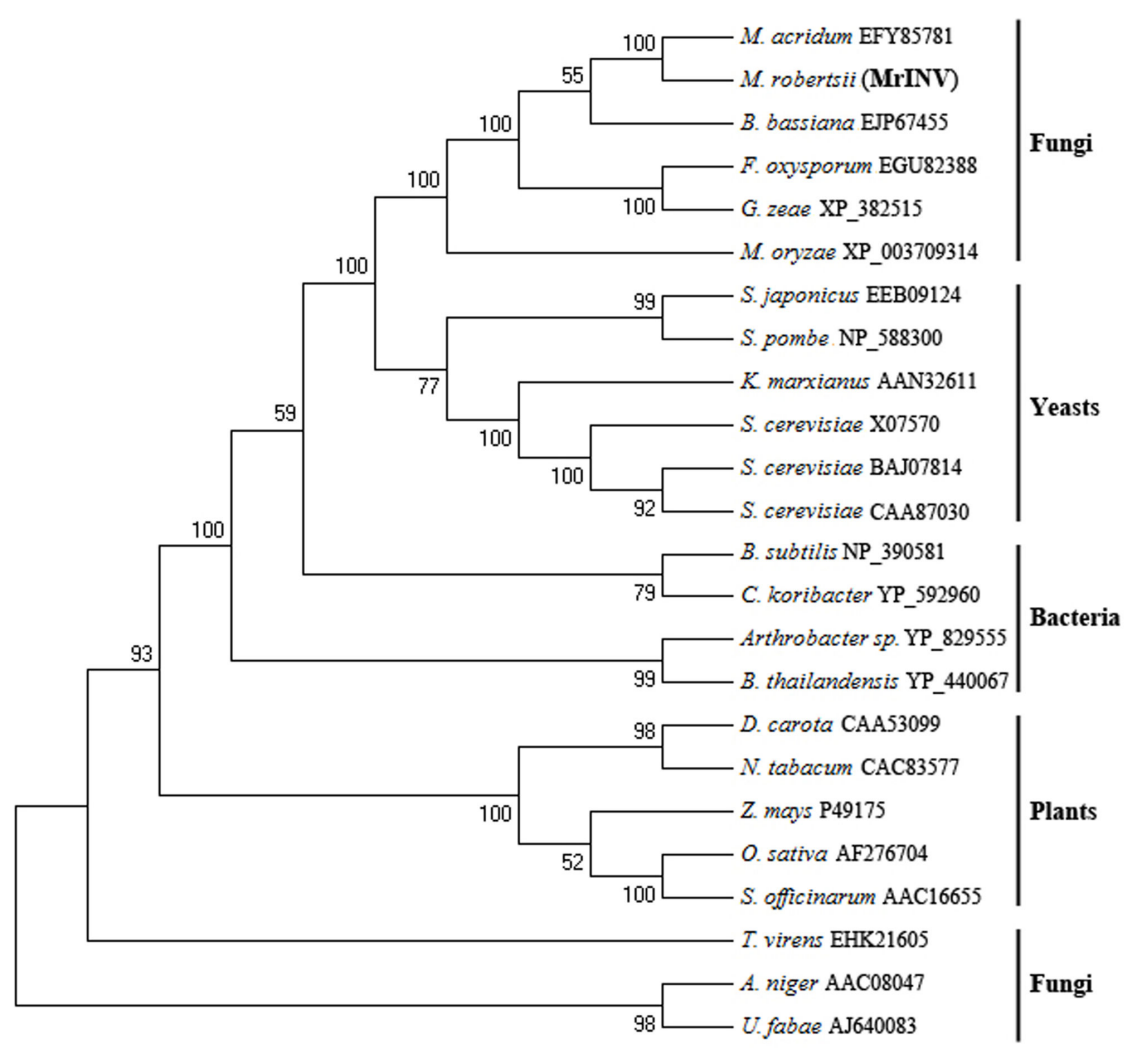
Neighbour-joining (NJ) tree of invertase from *M. robertsii* (MrINV) and other 23 invertases from bacteria, plants, yeasts and other fungi. Amino acid sequences were aligned using the ClustalX algorithm and the NJ tree was constructed with the MEGA4 software. Bootstrap values are adjacent to each internal node, representing the percertage of 1,000 bootsrap replicates.

Using an invertase TvINV from *T. virens* (accession no. EHK21605) as query, we identified a single homolog (*MrInv*) in the *M. robertsii* genome [14] with a maximum identity of 41% (5e^-7^). The Open Reading Frame (ORF) of *MrInv* is 1782-bp long coding for a putative 573 amino acid protein with a predicted molecular weight of 63.9 kDa, and is interrupted by one 60-bp intron. As deduced by SignalIP 4.1 [35], MrINV contains a predicted 24-aa signal peptide for secretion. The predicted cleavage site is between G24 and H25 and the calculated molecular weight of the mature protein is 61.3 kDa. The signal peptide resembled those on extracellular invertases from bacteria and some fungi including yeasts. Phylogenetic reconstruction grouped fungal invertases into two seperate clades (Figure 1). MrINV grouped with fungal invertases containing a NDPN box (conserved β-fructosidase motif) [36]. However, no homologous sequence was found in the scarab beetle specialist *M. majus*.

**Figure 2:**
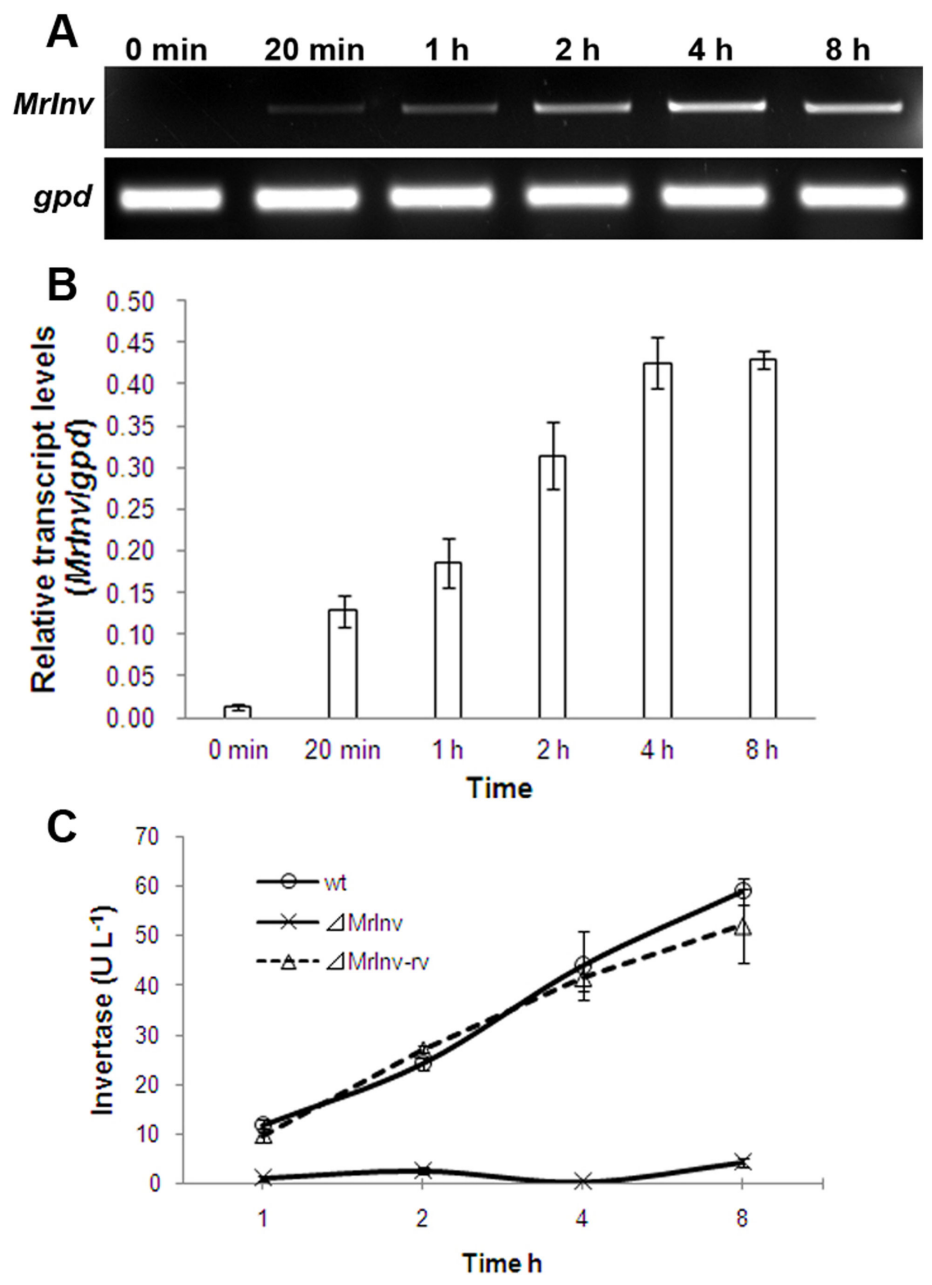
Characteristics of *MrInv* in *M. robertsii*. A, Expression assay with qPCR after mycelia transferred from SDB to minimal medium plus 1% sucrose, grown at 27 °C for 8 h. B, Relative transcript levels of *MrInv* gene versus the housekeeping *gpd* gene. *gpd*, glyceraldehyde-3-phosphate dehydrogenase gene. Values are means of three replicates with corresponding standard deviation. C, The invertase activity of *M. robertsii* 2575 wild-type strain, *⊿MrInv* and *⊿MrInv* revertant. Mycelia were transferred from SDB to minimal medium plus 1% sucrose, grown at 27 °C for 8 h. The filtrates from cultures were collected for enzymatic activity assay. Means are calculated from three replicates and bars represent standard error.

To better understand the role of *MrInv* during *M. robertsii* development, regulation of *MrInv* was determined under different cultural conditions using quantitative reverse transcription PCR (qPCR). Total RNA was isolated from mycelia grown on different carbohydrates including monosaccharides, homologous disaccharides and heterologous dioligosaccharides (Table 1). *MrInv* expression was up-regulated 40-fold in the presence of sucrose (Figure 2A), but other carbohydrates had no effect on expression (data not shown). A time-course for *MrInv* expression in sucrose culture showed onset at 20 minutes, and a peak at 4 h which then plateaud (Figure 2B).

**Table 1:** *In vitro* growth in different carbohydrates and root exudates.

	**wt**	**⊿*MrInv***	**⊿*MrInv-rv***
dH_2_O	1.3 ± 0.3	1.7 ± 0.3	1.5 ± 0.6
Sucrose	20.7 ± 1.2^A^	8.0 ± 1.2^B^	19.5 ± 0.9^A^
Raffinose	6.3 ± 0.9	6.7 ± 1.8	7.0 ± 0.5
Fructose	44.0 ± 2.0	45.0 ± 2.3	42.9 ± 1.1
Glucose	47.1 ± 1.1	45.8 ± 1.8	46.5 ± 1.3
Maltose	6.7 ± 1.9	9.0 ± 1.7	8.1 ± 1.2
NaNO_3_	8.7 ± 0.3	9.7 ± 0.9	9.2 ± 1.0
NaNO_3_ + Sucrose	19.7 ± 1.9^A^	10.0 ± 0.6^B^	19.1 ± 1.4^A^
NaNO_3_ + Raffinose	12.0 ± 0.6	12.0 ± 1.5	11.3 ± 0.9
NaNO_3_ + Fructose	47.3 ± 3.8	46.3 ± 5.6	45.9 ± 2.3
NaNO_3_ + Glucose	46.8 ± 0.9	46.5 ± 2.1	47.9 ± 1.7
NaNO_3_ + Maltose	13.3 ± 0.3	14.7 ± 1.3	15.0 ± 1.3
**Root exudates**			
1 mg mL^-1^	98.0 ± 0.6	98.3 ± 0.7	99.0 ± 1.0
0.1 mg mL^-1^	79.0 ± 0.6^A^	66.3 ± 2.7^B^	80.9 ± 1.8^A^
0.01 mg mL^-1^	51.3 ± 2.3^A^	22.3 ± 1.2^B^	50.1 ± 1.5^A^

Upper-case letters represent means statistically different at the 0.01 level.

### *MrInv* Disruption Suppressed M. *robertsii* Growth on Sucrose

To confirm that MrINV is a functional invertase involved in sucrose metabolism in *M. robertsii*, we produced a null mutant (*⊿MrInv*), deletion of the wild-type allele. To complement *⊿MrInv*, the genomic fragment of *MrInv* was transformed into *⊿MrInv* resulting in *⊿MrInv-rv*. The deletion of *MrInv* in *⊿MrInv* was confirmed by PCR using the genomic DNA as template (Figure S1). RT-PCR confirmed there was no *MrInv* expression in *⊿MrInv*, and as expected, *⊿MrInv* showed no invertase activity during 8 h growth on sucrose. Invertase activity was detected in the wild-type strain and ⊿*MrInv* revertant (⊿*MrInv-rv*) (Figure 2C), confirming that *MrInv* encodes the invertase up-regulated in the presence of sucrose.

**Figure 3:**
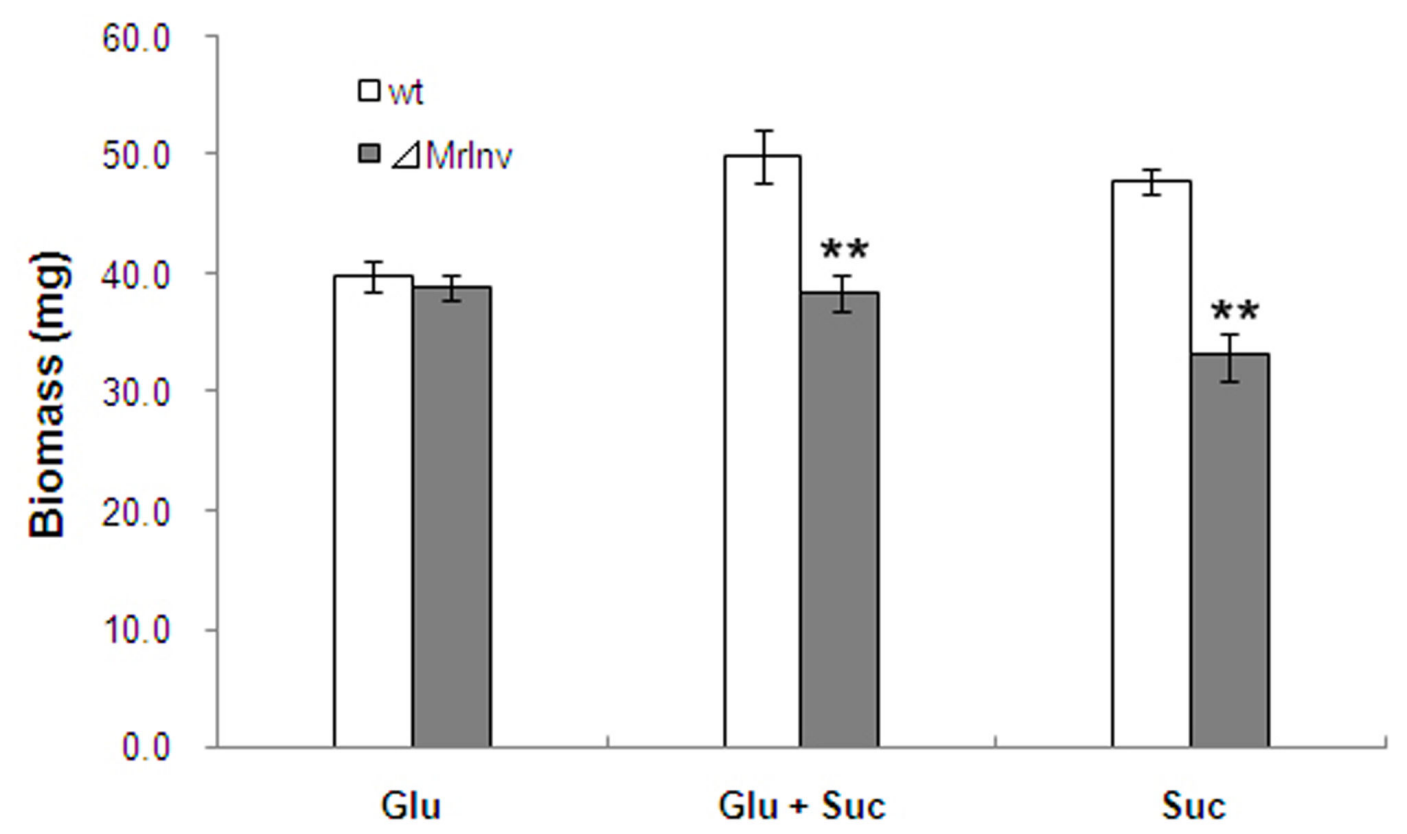
Biomass production of *M. robertsii* 2575 wild-type strain and ⊿*MrInv* in the presence of glucose and/or sucrose. Mycelia were transferred from SDB to minimal liquid medium supplemented with 1% glucose (Glu) and (Glu+Suc)/or 1% sucrose (Suc), grown at 27 °C for 12 h. Means were calculated from 3 replicates with corresponding standard errors. Experiments were repeated twice. ** indicates means statistically different at the 0.01 level.

To determine the role of *MrInv*, the wild-type and *⊿MrInv* strains were compared in colony morphology, germination rate and growth. Conidial germination of *⊿MrInv* (8.0% ± 1.2%) after 24 h in sucrose medium was reduced by 61% compared to the wild-type strain (20.7% ± 1.2%) (Table 1, *P* < 0.01). Likewise, colony growth (Figure S2) and biomass in sucrose medium were significantly reduced in *⊿MrInv* compared to the wild-type strain (Figure 3, *P* < 0.01). The addition of an inorganic nitrogen source (NaNO_3_) did not affect the ability of *MrInv* to utilize sucrose, and *⊿MrInv* and the wild-type strain germinated at similar rates in other carbohydrates, including glucose (Table 1). In addition,*⊿MrInv* grew at a similar rate as the wild-type strain using glucose as sole carbon source (Figure S3). Thus, the only impairment of*⊿MrInv* is in its ability to utilize sucrose which confirms MrINV is a functional invertase.

**Figure 4:**
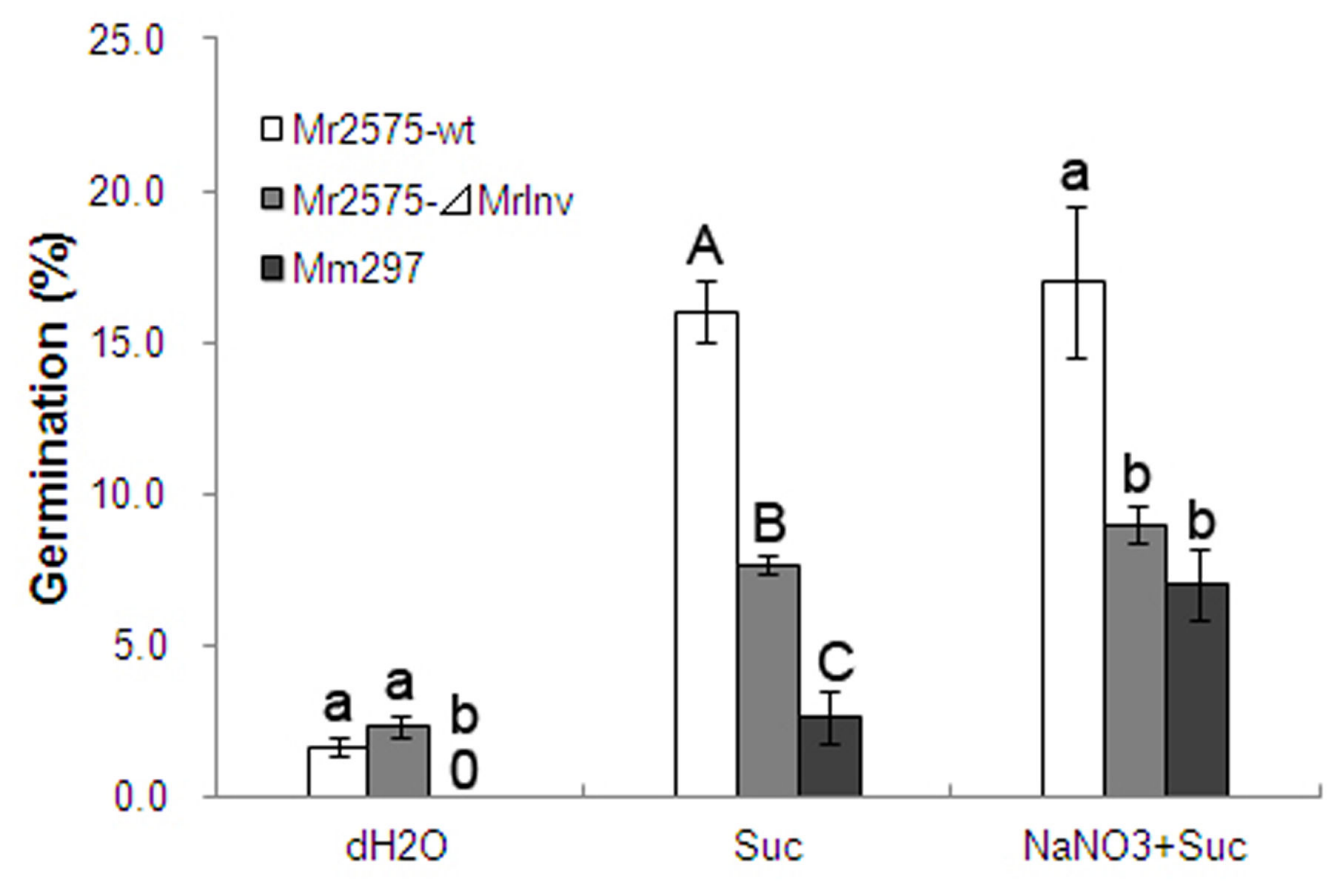
Conidial germination of *M. robertsii* 2575 wild-type strain (Mr2575-wt), *MrInv* disruption mutant (Mr2575-⊿MrInv) and *M. majus* 297 wild-type strain (Mm297). Conidia were cultured in sterile distilled water (dH2O), 0.1% sucrose (Suc) or 0.1% sucrose plus 0.1% NaNO_3_ (NaNO3+Suc). Conidial germination was determined after 24 h incubation at 27 °C. Means were calculated from 5 replicates. Bars represent the standard error. Upper-case and lower-case letters indicate means statistically different at the 0.01 and 0.05 level.

To determine whether MrINV is critical for sucrose utilization, the germination rate of *⊿MrInv* and *M. robertsii* wild-type on sucrose was compared with *M. majus* (lacks an invertase homolog). After 24 h incubation, *M. majus* showed a significnatly lower germination rate (2.3% ± 0.3%) on sucrose compared with *⊿MrInv* (7.7% ± 0.3%) and *M. robertsii* wild-type (16.0% ± 1.0%) (Figure 4, *P* < 0.01). Combining sucrose with a nitrogen source (NaNO_3_), elevated *M. majus* germination (7.0% ± 1.2%) to a similar level as*⊿MrInv* (9.0% ± 0.6%) but still lower than *M. robertsii* wild-type (17.0% ± 2.5%) (Figure 4, *P* < 0.05). Though lacking invertase, *⊿MrInv* and *M. majus* strains can still grow poorly on sucrose as sole carbon source which suggests that *Metarhizium* spp. has additional less efficient mechanisms for metabolizing sucrose for growth and development. This contrasts with *T. virens* as invertase is its sole means of utilizing sucrose [22].

### *MrInv* Disruption Impaired M. *robertsii* Rhizosphere Competency but Enhanced Root Colonization

To analyze the involvement of *MrInv* in the rhizosphere competency of *M. robertsii*, we investigated fungal survivorship in the soil and measured root colonization. Firstly, to mimic rhizospheric nutrient sources we used soybean root exudate, which contains plant-derived sucrose as a major component [31]. No difference in the germination rate was observed between *⊿MrInv* and wild-type strains at high concentration of root exudate (1 mg mL^-1^). But at low concentrations (0.1 mg mL^-1^ and 0.01 mg mL^-1^), germination of *⊿MrInv* was reduced by16% and 56%, respectively (Table 1, *P*< 0.01). 

**Figure 5:**
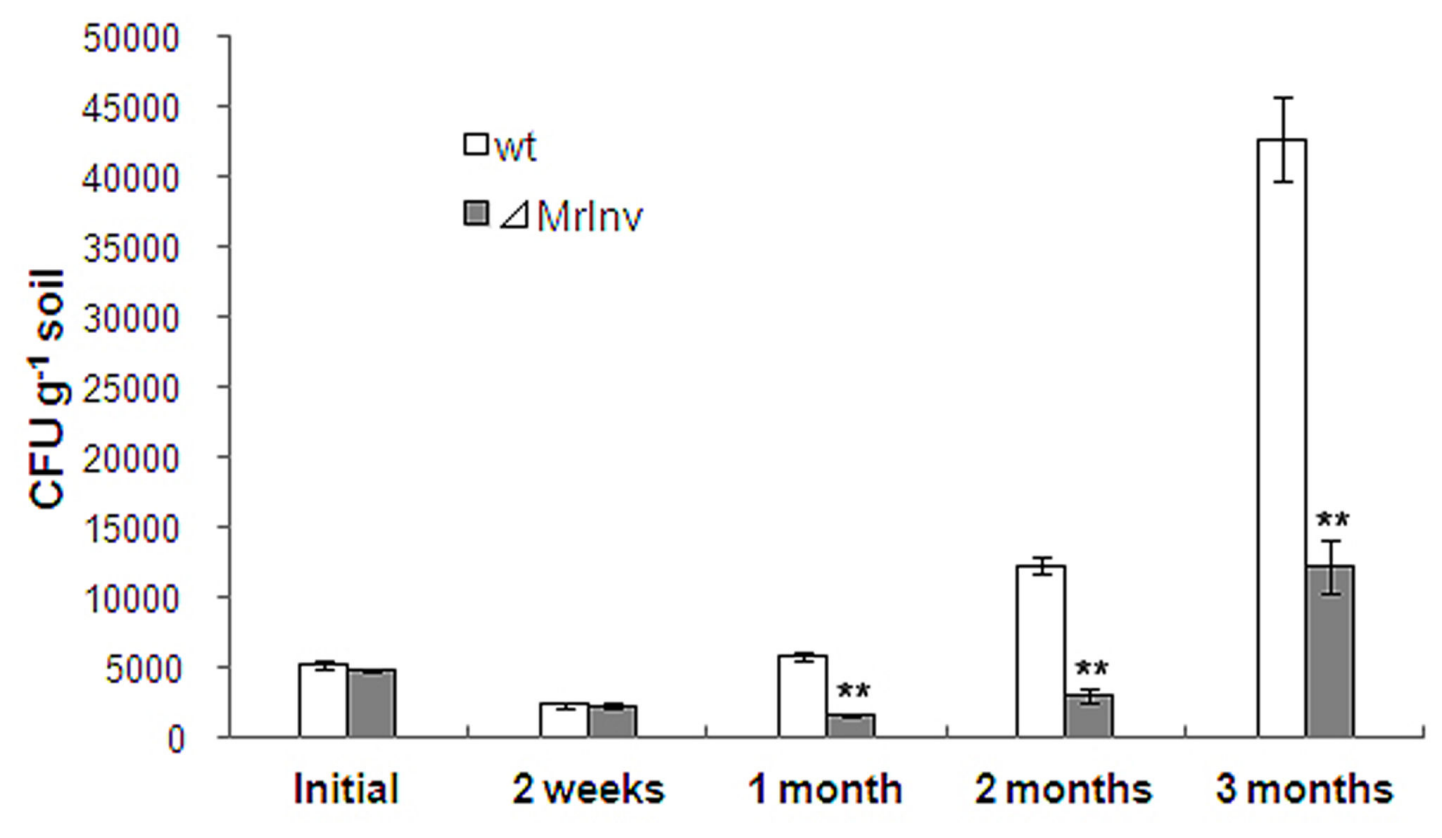
Rhizosphere competency of *M. robertsii* wild-type strain (wt) and *MrInv* disruption mutant (⊿MrInv). Switchgrass seeds were inoculated with *M. robertsii* spores and grown in the growth chamber at 25 °C with 14:10 h light:dark cycle. Rhizospheric populations of each strain were measured by counting the number of CFUs in rhizospheric soils. Initial indicates the number of CFUs from each treatment immediately after inoculation. Means were calculated from nine replicates. Bars represent the standard error. ** indicates means statistically different at the 0.01 level.

The poor growth of *⊿MrInv* in root exudate implied that *⊿MrInv* may be impaired in rhizosphere competency. To investigate rhizospheric interactions and fungal root colonization, spores of *⊿MrInv* and wild-type strains were inoculated into soil microcosms containing switchgrass. Switchgrass is easy to culture in lab conditions and is a well characterized host for *M. robertsii* [32]. The rhizosphere competency of *⊿MrInv* and wild-type strains was determined by counting colony-forming units (CFUs) in soil samples [30]. Initial densities were determined by counting CFUs immediately after adding fungal spores. Two weeks post-inoculation the number of *⊿MrInv* and wild-type CFUs had dropped by half. Two months post-inoculation, *⊿MrInv* levels were still 37% less than the initial density, but the number of wild-type CFUs had increased 2.4-fold. At three months,*⊿MrInv* and wild-type rhizospheric populations were 2.5-fold and 8-fold higher, respctively. Thus, compared to the wild-type, the number of *⊿MrInv* CFUs were 72%, 75% and 71% less in the first, second and third month post-inoculation, respectively (Figure 5, *P* < 0.01). CFU counts of *⊿MrInv* and wild-type strains in bulk soil (soil containing fungi but no seeds) declined at the same rate over 3 months. The significant reduction of *⊿MrInv* in the rhizosphere microcosms relative to the wild-type suggests *MrInv* is important for rhizosphere competency through utilization of plant-derived sucrose as carbon source.

**Figure 6:**
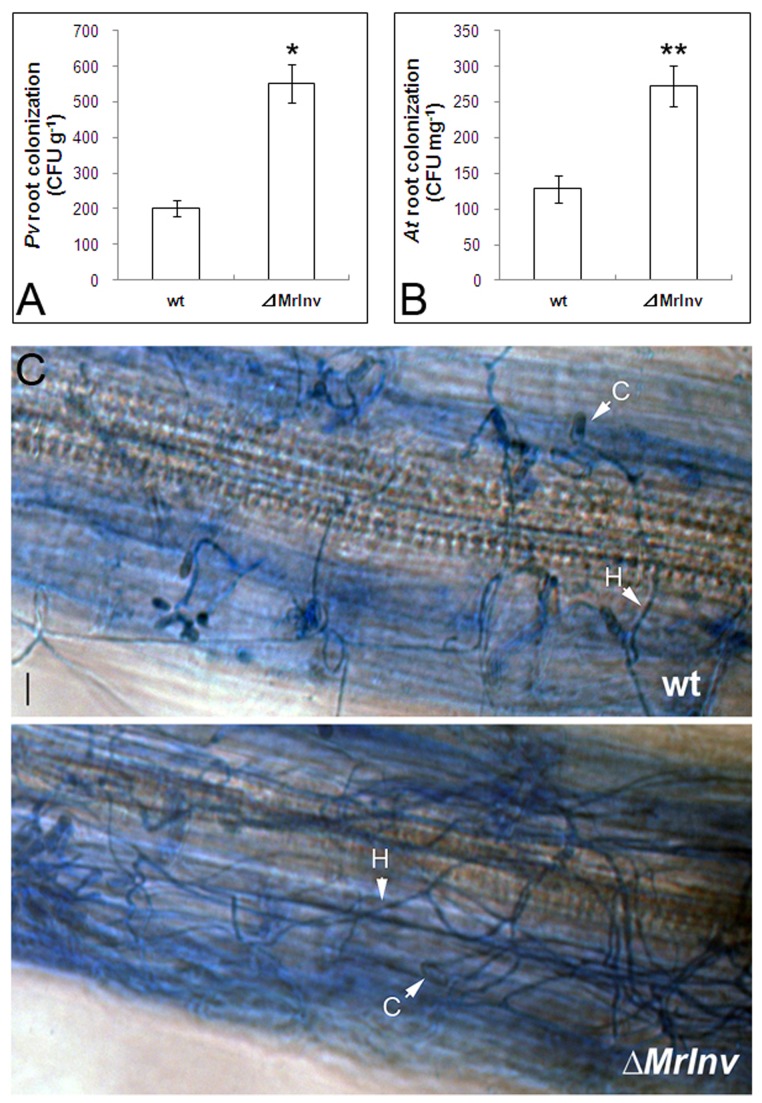
Root colonization of switchgrass (Pv) and *A. thaliana* (At) by *M. robertsii* wild-type strain (wt) and *MrInv* disruption mutant (⊿MrInv). A, 3-month-old switchgrass roots from the soils containing *M. robertsii* strains were collected for root colonization assay. B, *A. thaliana* seedlings were inoculated with *M. robertsii* spores from each strain in the hydroponic system. After 48 h incubation, *A. thaliana* roots were collected for root colonization assay. Means are calculated from 9 (Pv) or 27 (At) replicates of each treatment and bars represent the standard error. ** and * indicate means statistically different at the 0.01 and 0.05 level. C, Photographs of *A. thaliana* root colonization by *M. robertsii* wild-type strain (top) and *⊿MrInv* (bottom) after 48 h incubation. C, conidium; H, hypha. Scale bar = 5 μm.

As well as colonizing the rhizosphere, *M. robertsii* also grows on root surfaces [30]. Since deletion of *MrInv* reduced rhizosphere competency, we also determined if *MrInv* facilitates colonization of roots by counting CFUs extracted from switchgrass roots. Deleting *MrInv* produced a 2.7-fold increase in root colonization relative to the wild-type strain (Figure 6A, P < 0.05). To determine if the increased root colonization by *⊿MrInv* on switchgrass is applicable to other plants, *A. thaliana* roots were inoculated with *⊿MrInv* or wild-type strain in a hydroponic system. Root colonization was determined after 48 h incubation by counting CFUs. Colonization of *A. thaliana* roots by *⊿MrInv* was increased 2.1-fold relative to the wild-type strain (Figure 6B, P < 0.01). Microscopic observation confirmed that *⊿MrInv* hyphae proliferated more than wild-type hyphae on *A. thaliana* roots (Figure 6C).

### *MrInv* Disruption reduces Carbon Catabolite Repression and Increases Hydrolytic Enzyme Activity

**Figure 7:**
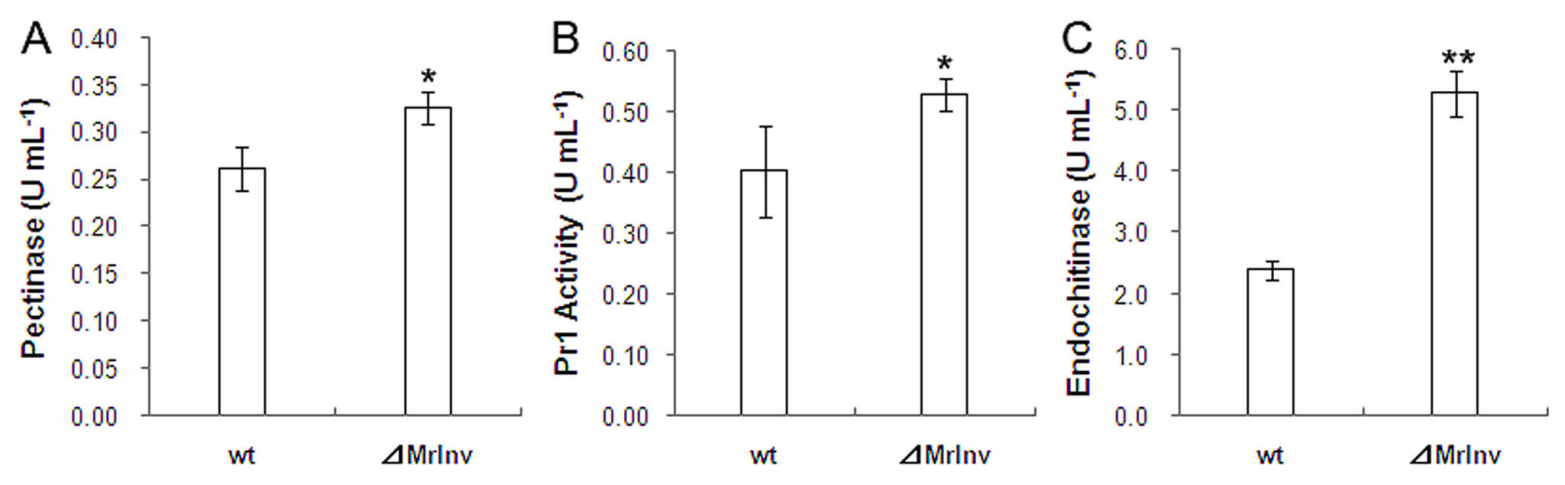
The activity assay of hydrolytic enzymes produced by *M. robertsii* 2575 wild-type strain and *⊿MrInv*. The filtrates from cultures of each strain grown with *A. thaliana* seedlings for 48 h were collected, and the enzymatic activity of pectinase (A), subtilisin Pr1 (B) and endochitinase (C) were measured. Means are calculated from three replicates and bars represent standard deviation. Similar results were obtained in two independent experiments. ** and * indicate means statistically different at the 0.01 and 0.05 level.

During *Metarhizium*-plant associations, hyphae penetrate the superficial cell layers [30]. To breach the epidermic cell wall, fungi secrete hydrolytic enzymes such as pectinase [37]. Likewise, entomopathogenic fungi produce cuticle degrading proteinases and endochitinases that target the integuments of their insect hosts [38-40]. We compared production of hydrolytic enzymes by the *⊿MrInv* and wild-type strains when colonizing roots. Higher levels of pectinase (Figure 7A, P < 0.05), Pr1 (Figure 7B, P < 0.05) and endochitinase (Figure 7C, P < 0.01) activities were produced by *⊿MrInv* in the hydroponic system containing *A. thaliana* seedlings.

**Figure 8:**
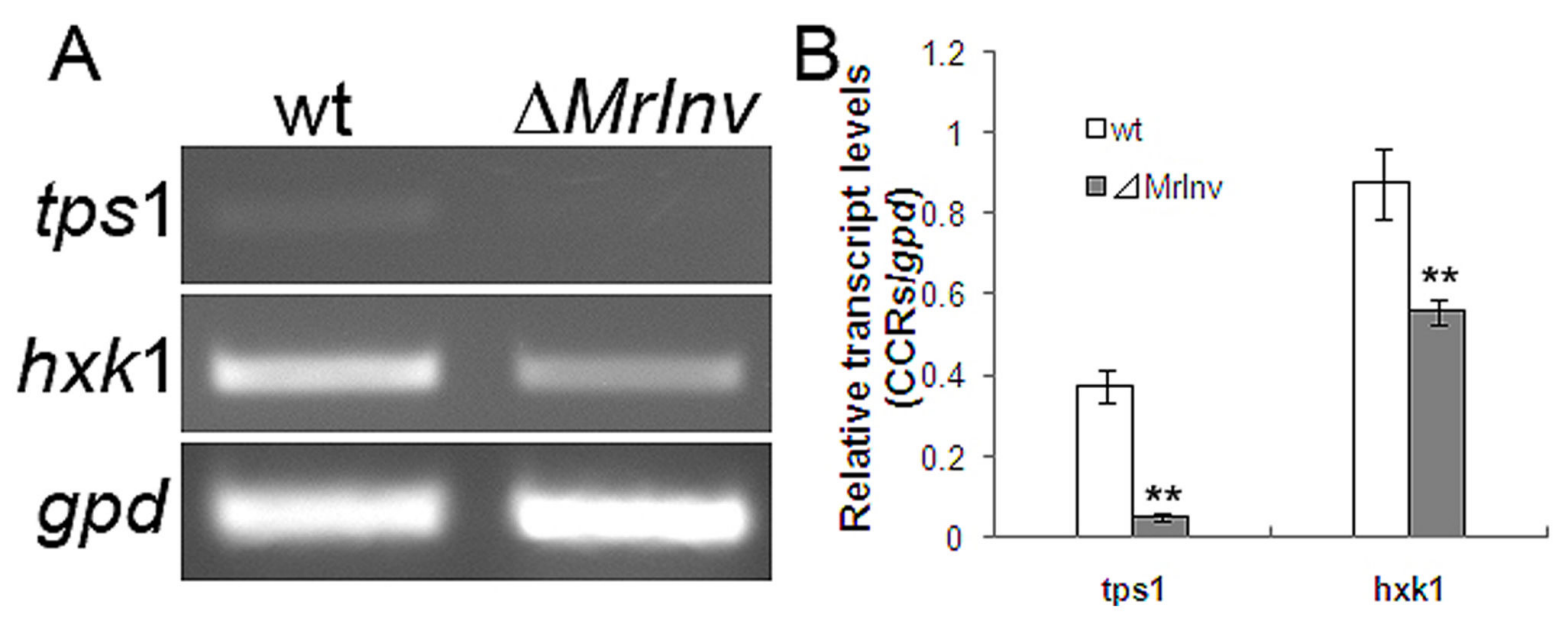
Expression of CCR-related genes in *M. robertsii* 2575 wild-type strain and *ΔMrInv* in the presence of sucrose. A, Semi-quantitative RT-PCR analysis of CCR-related genes. Mycelia were transferred from SDB to minimal medium plus 1% sucrose, grown at 27 °C for 30 min. Total RNA was extracted from fungal biomass and qPCR was performed. B, Relative transcript levels of CCR-related genes versus the housekeeping *gpd* gene. *tps*1, trehalose-6-phosphate synthase gene; *hxk*1, hexose kinase gene; *gpd*, glyceraldehyde-3-phosphate dehydrogenase gene. Values are means of three replicates with corresponding standard deviation. ** indicates means statistically different at the 0.01 level.

Production of many hydrolytic enzymes in fungi is regulated by carbon catabolite repression (CCR) [41]. We hypothesized that hydrolytic enzymes are up-regulated in *⊿MrInv* due to less glucose uptake and thereby reduced CCR. To test this we measured the expression of CCR-related genes. Consistent with greater CCR, sucrose induced higher levels of the CCR-related genes *tps*1 (8.3-fold) and *hxk*1 (2.6-fold) in the wild-type than in *⊿MrInv* (Figure 8, *P* < 0.01). The current study suggests that hydrolytic enzymes from *M. robertsii* are involved in the root colonization process and the hydrolytic enzyme activities in *MrInv* deficiency strain are up-regulated possibly through the release of CCR.

### Plant growth

The growth of switchgrass inoculated with *⊿MrInv* and wild-type strains was monitored for 3 months at one month intervals to determine whether *MrInv* expression had an impact on plant growth. No differences were found between plants inoculated with *⊿MrInv* and wild-type strain in stem length, leaf chlorophyll content, root length and plant biomass (Figure S4). In addition, the growth of switchgrass uninoculated with either fungi was similar with the plants inoculated with *M. robertsii*.

## Discussion

Enormous numbers of microbes inhabit the rhizosphere using various products of plant photosynthesis, and in return some microbes boost plant growth and health through several mechanisms including phytostimulation, biofertilisation, bioremediation and biological control [42]. The rhizosphere competence of *M. robertsii* was discovered comparatively recently in a field plot of cabbages [5]. *M. robertsii* is the only *Metarhizium* species found to associate with grass roots in the field [32], but in this study, we found that both switchgrass and the dicotyledonous *A. thaliana* supported extensive root colonization by *M. robertsii*. 

Compared to interactions between fungal pathogens and their hosts, little is known about the molecular mechanisms underlying the complex interactions between roots, root exudate and any rhizospheric fungus, including the best studied examples in the genus *Trichoderma*. It is particularly important to comprehensively understand the natural ecological role of *Metarhizium* and *Trichoderma* in order to optimize their use as biological control agents. Table 2 summarizes currently understood similarities and differences between *Metarhizium* and *Trichoderma*. Each fungus has evolved its own multi-faceted and robust mechanisms to overcome the challenges encountered on plant roots.

**Table 2:** Commonalities and differences between *Metarhizium* and *Trichoderma* in root colonization.

**Genus**	***Metarhizium***	***Trichoderma***
**Pathogen**	Of insects	Of fungi (some strains)
**Root colonization**	Minority of strains	Minority of strains
**Beneficial effects**	Inhibit plant pathogens [7]	Inhibit plant pathogens [10]
	Enhancing the uptake of micronutrients [8]	Enhancing the uptake of micronutrients [48]
	Translocating nitrogen from killed insects to plants [9]	Facilitate plant resistance to abiotic stress [49]
	Killing soil insects [6]	Induction of plant defenses [44]
		Boosting plant growth by fungus-derived phytohormone [50]
**Adhere to root**	Adhesin (MAD2) [16]	Hydrophobin [15]
**Adhesive structure**	No specific adhesive structure	Form chlamydospore [51]
**Survival in the soil**	Survive adverse environmental conditions [52]	Inability to survive adverse environmental conditions [53]
**Nutrient sources**	Products of plant photosynthesis, particularly sugars	Products of plant photosynthesis, particularly sugars
**Sugars uptake**	Raffinose pump (MRT) [30]	
	Invertase (MrINV, **this study**)	Invertase (TvINV) [22]
**INV properties**	Acidic	Acidic
**INV Localization**	Extracellular	Intracellular
**INV Loss-of-**	Impairing rhizosphere competency	
**Function**	Increase root colonization	Increase root colonization
**Mechanism**	Upregulation of hydrolytic enzymes through carbon catabolite derepression	Upregulation of hydrolytic enzymes through carbon catabolite derepression

*In vitro* growth assays have shown that *M. robertsii* grows well in root exudates from soybeans and switchgrass [30,32]. In the current study, we show that *M. robertsii* uses MrINV to specifically hydrolyze sucrose to monosaccharides, and while *M. robertsii* utilizes a wide variety of carbohydrates the highest germination rates are on monosaccharides. *M. robertsii* can also take up sucrose using its unique oligosaccharide transporter (MRT) [30,32]. Disruption of *Mrt* resulted in reduced rhizosphere competency confirming a role for root-derived oligosaccharides in the symbiotic relationship between *M. robertsii* and plants [30]. However, germination and growth utilizing sucrose are reduced in the absence of the extracellular sucrose-induced MrINV. Given the complexity of carbon sources in root exudate, diverse strategies for utilizing sugars seems well attuned to *M. robertsii*’s opportunistic lifestyle and wide range of plant associations. 

A phylogenetic reconstruction classifies MrINV as a fungal secreted acidic invertase, and it clusters separately from non-secreted enzymes including the query sequence TvINV from *T. virens*. Blastp and genomic blast searches confirm that *MrInv* is a single copy gene in *M. robertsii*. The specialist beetle pathogen *M. majus* lacks a homolog of MrINV and does not grow on sucrose as sole carbon source. *M. majus* has not to date been identified as a root colonizer [32], suggesting that rhizospheric competence may be related to the sugar metabolism of different *Metarhizium* species.

*Metarhizium* is attracted to and associates intimately with root surfaces in the soil [32,43]. However, few studies have focused on the characteristics of *Metarhizium* interactions with roots as they appear under the microscope. In this study, the microscopic observation of the colonization of *A. thaliana* root by *M. robertsii* suggests the root invasion occurs by penetration of the epidermis and further ingress into the outer cortex, which share a similar pattern of colonization with *Trichoderma* [15]. However, unlike *Trichoderma*, we observed no specific adhesive structures during root colonization by *M. robertsii*. Sasan and Bidochka (2012) have reported that *M. robertsii* promotes plant root growth [43], and we have observed the same in field conditions when plants are growing under sub-optimum feeding and watering regimes (Unpubl. Data). The lack of impact on plant growth in the current study is probably because the plants were growing in a nutrient rich soil with adequate water.

The most significant finding of the current study is that disruption of *MrInv* reduced *M. robertsii*’s survival in the overlapping rhizospheres surrounding switchgrass roots, but significantly enhanced root colonization. *Trichoderma* lacking the intracellular *TvInv* also shows increased colonization of maize roots [22]. There is no information about *TvInv*’s effects on competency in soil but *Trichoderma* lacking *TvInv* showed increased expression of hydrolytic enzymes that weaken epidermal cell walls [44]. This could result from carbon catabolite derepression because of a reduction in glucose and related sugars in the mutant [45,46]. The *tps*1 (encoding trehalose-6-phosphate synthase) and *hxk*1 (encoding hexose kinase) are important mediators of CCR in fungi. They are induced by glucose and mediate glycolysis and carbon catabolite repression [47]. In our study, sucrose induced greater expression of *tps*1 and *hxk*1 in the wild-type than in *⊿MrInv* consistent with derepression in the mutant. *M. robertsii* invades insects by direct penetration of the cuticle facilitated by the production of a battery of extracellular enzymes, including proteinases (Pr1), chitinases and esterases [38]. The higher levels of Pr1 and endochitinase produced by *⊿MrInv* on *Arabidopsis* root probably result from carbon catabolite derepression in the mutant. In the field these enzymes could be involved in scavenging nutrients from fungi and insects in the soil since they do not target the plant cell wall. *⊿MrInv*’s upregulation of pectinase, which hydrolyzes a major polysaccharide substrate in plant cell walls [37], provides a direct connection between *Metarhizium*-produced hydrolytic enzymes and plant cell wall-degradation. Furthermore, increased colonization of monocotyledon (switchgrass) and dicotyledon (*A. thaliana*) roots suggests that the effect is irrespective of the plant species. In spite of increased production of cell wall-degrading enzymes *⊿MrInv*’s competency in the soil is sharply reduced suggesting that at a small distance from the roots sucrose is a more important source of nutrients than polymers.

## Supporting Information

Figure S1 **Verification of *MrInv* disruption and complement in *M. robertsii* 2575.** A, The schematic diagram of DNA crossover and integration in the genome of wild-type and mutant strains. B, wt, wild-type stain; ⊿*MrInv*, the mutant in which *MrInv* was replaced with the bar selective marker by homologous recombination; ⊿*MrInv*-*rv*, a transformant in which the ⊿*MrInv* complemented by the *MrInv* genomic fragment.
(PDF)

Figure S2 **Growth of *M. robertsii* 2575 wild-type strain (wt) and *MrInv* disruption mutant (⊿*MrInv*) on minimal medium agar plates supplemented with 1% sucrose at 27 °C for 10 d. ⊿*MrInv* grew comparatively less well than wild-type strain on sucrose.**
(PDF)

Figure S3 **Growth of *M. robertsii* 2575 wild-type strain (wt) and *MrInv* disruption mutant (⊿*MrInv*) on PDA (top) for 5 d and M100 (bottom) agar plates at 27 °C for 10 d.** Scale bar = 1 cm.
(PDF)

Figure S4 **Switchgrass growth was monitored by measuring the shoot length and leaf chlorophyll content at one month intervals post-inoculation.** Switchgrass were harvested after three months. The root length and plant dry biomass were determined. Nine plants from each pot were randomly selected for measurement. Values are means calculated from 27 replicates and bars represent the standard error.
(PDF)

Table S1 **Primers used in this study.**
(PDF)

## References

[1] Bischoff JF, Rehner SA, Humber RA (2009) A multilocus phylogeny of the *Metarhizium* *anisopliae* lineage. Mycologia 101: 512-530. doi:10.3852/07-202. PubMed: 19623931. 19623931

[2] Lomer CJ, Bateman RP, Johnson DL, Langewald J, Thomas M (2001) Biological control of locusts and grasshoppers. Annu Rev Entomol 46: 667-702. doi:10.1146/annurev.ento.46.1.667. PubMed: 11112183. 11112183

[3] Maniania NK, Sithanantham S, Ekesi S, Ampong-Nyarko K, Baumgärtner J et al. (2003) A field trial of the entomogenous fungus *Metarhizium* *anisopliae* for control of onion thrips, *Thrips* *tabaci* . Crop Protect 22: 553-559. doi:10.1016/S0261-2194(02)00221-1.

[4] Shah PA, Pell JK (2003) Entomopathogenic fungi as biological control agents. Appl Microbiol Biotechnol 61: 413-423. PubMed: 12764556. 12764556 10.1007/s00253-003-1240-8

[5] Hu G, St Leger RJ (2002) Field Studies Using a Recombinant Mycoinsecticide (Metarhizium anisopliae) Reveal that It Is Rhizosphere Competent. Appl Environ Microbiol 68: 6383-6387. doi:10.1128/AEM.68.12.6383-6387.2002. PubMed: 12450863. 12450863 PMC134390

[6] Kabaluk JT, Ericsson JD (2007) Seed treatment increases yield of field corn when applied for wireworm control. Agron J 99: 1377-1381. doi:10.2134/agronj2007.0017N.

[7] Ownley B, Gwinn K, Vega F (2010) Endophytic fungal entomopathogens with activity against plant pathogens: ecology and evolution. BioControl 55: 113-128. doi:10.1007/s10526-009-9241-x.

[8] O'Brien T (2009) The saprophytic life of *Metarhizium* *anisopliae*. PhD thesis. College Park: University of Maryland.

[9] Behie SW, Zelisko PM, Bidochka MJ (2012) Endophytic insect-parasitic fungi translocate nitrogen directly from insects to plants. Science 336: 1576-1577. doi:10.1126/science.1222289. PubMed: 22723421. 22723421

[10] Harman GE (2006) Overview of Mechanisms and Uses of *Trichoderma* spp. Phytopathology 96: 190-194. doi:10.1094/PHYTO.2006.96.6.S190. PubMed: 18943924. 18943924

[11] Lorito M, Woo SL, Harman, Gary E, Monte E (2010) Translational Research on *Trichoderma*: From 'Omics to the Field. Annu Rev Phytopathol 48: 395-417. doi:10.1146/annurev-phyto-073009-114314. PubMed: 20455700. 20455700

[12] Faria M. Rd, Wraight SP (2007); Mycoinsecticides, Mycoacaricides A comprehensive list with worldwide coverage and international classification of formulation types. Biol Contr 43: 237-256. doi:10.1016/j.biocontrol.2007.08.001.

[13] Mukherjee PK, Buensanteai N, Moran-Diez ME, Druzhinina IS, Kenerley CM (2012) Functional analysis of non-ribosomal peptide synthetases (NRPSs) in *Trichoderma* *virens* reveals a polyketide synthase (PKS)/NRPS hybrid enzyme involved in the induced systemic resistance response in maize. Microbiology 158: 155-165. doi:10.1099/mic.0.052159-0. PubMed: 22075027. 22075027

[14] Gao Q, Jin K, Ying S-H, Zhang Y, Xiao G et al. (2011) Genome Sequencing and Comparative Transcriptomics of the Model Entomopathogenic Fungi *Metarhizium* *anisopliae* and *M*. *acridum* . PLOS Genet 7: e1001264. 21253567 10.1371/journal.pgen.1001264 PMC3017113

[15] Viterbo ADA, Chet I (2006): TasHyd1, a new hydrophobin gene from the biocontrol agent *Trichoderma**asperellum*, is involved in plant root colonization. Molecular Plant Pathology 7: 249-258 10.1111/j.1364-3703.2006.00335.x 20507444

[16] Wang C, St Leger RJ (2007) The MAD1 adhesin of *Metarhizium* *anisopliae* links adhesion with blastospore production and virulence to insects, and the MAD2 adhesin enables attachment to plants. Eukaryot Cell 6: 808-816. doi:10.1128/EC.00409-06. PubMed: 17337634. 17337634 PMC1899246

[17] Baudoin E, Benizri E, Guckert A (2003) Impact of artificial root exudates on the bacterial community structure in bulk soil and maize rhizosphere. Soil Biol Biochem 35: 1183-1192. doi:10.1016/S0038-0717(03)00179-2.

[18] Jaeger CH, Lindow SE, Miller W, Clark E, Firestone MK (1999) Mapping of sugar and amino acid availability in soil around roots with bacterial sensors of sucrose and tryptophan. Appl Environ Microbiol 65: 2685-2690. PubMed: 10347061. 10347061 10.1128/aem.65.6.2685-2690.1999 PMC91396

[19] Mahmood T, Woitke M, Gimmler H, Kaiser WM (2002) Sugar exudation by roots of kallar grass [*Leptochloa* *fusca* (L.) Kunth] is strongly affected by the nitrogen source. Planta 214: 887-894. doi:10.1007/s00425-001-0697-x. PubMed: 11941465. 11941465

[20] Chaudhuri A, Bharadwaj G, Maheshwari R (1999) An unusual pattern of invertase activity development in the thermophilic fungus *Thermomyces* *lanuginosus* . FEMS Microbiol Lett 177: 39-45. doi:10.1111/j.1574-6968.1999.tb13711.x. PubMed: 10475745. 10475745

[21] Rubio MC, Navarro AR (2006) Regulation of invertase synthesis in *Aspergillus* *niger* . Enzyme Microb Technol 39: 601-606. doi:10.1016/j.enzmictec.2005.11.011.

[22] Vargas WA, Mandawe JC, Kenerley CM (2009) Plant-derived sucrose is a key element in the symbiotic association between *Trichoderma* *virens* and maize plants. Plant Physiol 151: 792-808. doi:10.1104/pp.109.141291. PubMed: 19675155. 19675155 PMC2754623

[23] Voegele RT, Wirsel S, Möll U, Lechner M, Mendgen K (2006) Cloning and characterization of a novel invertase from the obligate biotroph *Uromyces* *fabae* and analysis of expression patterns of host and pathogen invertases in the course of infection. Mol Plant Microbe Interact 19: 625-634. doi:10.1094/MPMI-19-0625. PubMed: 16776296. 16776296

[24] Sauer DB, Burroughs R (1986) Disinfection of seed surfaces with sodium hypochlorite. Phytopathology 76: 745-749. doi:10.1094/Phyto-76-745.

[25] Miché L, Balandreau J (2001) Effects of rice seed surface sterilization with hypochlorite on inoculated *Burkholderia* *vietnamiensis* . Appl Environ Microbiol 67: 3046-3052. doi:10.1128/AEM.67.7.3046-3052.2001. PubMed: 11425720. 11425720 PMC92979

[26] Fang W, Pei Y, Bidochka MJ (2006) Transformation of *Metarhizium* *anisopliae* mediated by *Agrobacterium* *tumefaciens* . Can J Microbiol 52: 623-626. doi:10.1139/w06-014. PubMed: 16917517. 16917517

[27] Liao X-g, Fang W-g, Zhang Y-j, Fan Y-h, Wu X-w et al. (2008) Characterization of a highly active promoter, PBbgpd, in *Beauveria* *bassiana* . Curr Microbiol 57: 121-126. doi:10.1007/s00284-008-9163-3. PubMed: 18443858. 18443858

[28] Lin L, Wang F, Wei D (2011) Chlorimuron ethyl as a new selectable marker for disrupting genes in the insect-pathogenic fungus *Metarhizium* *robertsii* . J Microbiol Methods 87: 241-243. doi:10.1016/j.mimet.2011.07.018. PubMed: 21851837. 21851837

[29] Fang W, Pava-ripoll M, Wang S, St Leger R (2009) Protein kinase A regulates production of virulence determinants by the entomopathogenic fungus, *Metarhizium* *anisopliae* . Fungal Genet Biol 46: 277-285. doi:10.1016/j.fgb.2008.12.001. PubMed: 19124083. 19124083

[30] Fang W, St Leger RJ (2010) *Mrt*, a gene unique to fungi, encodes an oligosaccharide transporter and facilitates rhizosphere competency in *Metarhizium* *robertsii* . Plant Physiol 154: 1549-1557. doi:10.1104/pp.110.163014. PubMed: 20837701. 20837701 PMC2971628

[31] Pava-Ripoll M, Angelini C, Fang W, Wang S, Posada FJ et al. (2011) The rhizosphere-competent entomopathogen Metarhizium anisopliae expresses a specific subset of genes in plant root exudate. Microbiology 157: 47-55. doi:10.1099/mic.0.042200-0. PubMed: 20947574. 20947574

[32] Wyrebek M, Huber C, Sasan RK, Bidochka MJ (2011) Three sympatrically occurring species of *Metarhizium* show plant rhizosphere specificity. Microbiology 157: 2904-2911. doi:10.1099/mic.0.051102-0. PubMed: 21778205. 21778205

[33] Viterbo A, Harel M, Horwitz BA, Chet I, Mukherjee PK (2005) *Trichoderma* mitogen-activated protein kinase signaling is involved in induction of plant systemic resistance. Appl Environ Microbiol 71: 6241-6246. doi:10.1128/AEM.71.10.6241-6246.2005. PubMed: 16204544. 16204544 PMC1266020

[34] St Leger RJ, Charnley AK, Cooper RM (1987) Characterization of cuticle-degrading proteases produced by the entomopathogen *Metarhizium* *anisopliae* . Arch Biochem Biophys 253: 221-232. doi:10.1016/0003-9861(87)90655-2. PubMed: 3545084. 3545084

[35] Petersen TN, Brunak S, Heijne (2011) Gv, Nielsen H. Signal: 4 0: discriminating signal peptides from transmembrane regions. Nature Methods 8: 785-786

[36] Goetz M, Roitsch T (2000) Identification of amino acids essential for enzymatic activity of plant invertases. J Plant Physiol 157: 581-585. doi:10.1016/S0176-1617(00)80115-7.

[37] Prade RA, Ayoubi P, Zhan D, Mort AJ (1999) Pectins, pectinases and plant-microbe interactions. Biotechnol Genet Eng Rev 16: 361-392. doi:10.1080/02648725.1999.10647984. PubMed: 10819085. 10819085

[38] Clarkson JM, Charnley AK (1996) New insights into the mechanisms of fungal pathogenesis in insects. Trends Microbiol 4: 197-203. doi:10.1016/0966-842X(96)10022-6. PubMed: 8727600. 8727600

[39] Herrera-Estrella A, Chet I (1999) Chitinases in biological control. In: Jollès P Muzzarelli RAA Chitin and Chitinases. Basel: Birkhäuser Verlag pp. 171-184. 10.1007/978-3-0348-8757-1_12 10906959

[40] Samuels RI, Paterson IC (1995) Cuticle degrading proteases from insect moulting fluid and culture filtrates of entomopathogenic fungi. Comp Biochem Physiol B Biochem Mol Biol 110: 661-669. doi:10.1016/0305-0491(94)00205-9. PubMed: 7749618. 7749618

[41] Ruijter GJG, Visser J (1997) Carbon repression in *Aspergilli* . FEMS Microbiol Lett 151: 103-114. doi:10.1111/j.1574-6968.1997.tb12557.x. PubMed: 9228741. 9228741

[42] Weert S, Bloemberg G (2006) Rhizosphere competence and the role of root colonization in biocontrol. In: Gnanamanickam S Plant-Associated Bacteria. Netherlands: Springer Verlag pp. 317-333.

[43] Sasan RK, Bidochka MJ (2012) The insect-pathogenic fungus *Metarhizium* *robertsii* (Clavicipitaceae) is also an endophyte that stimulates plant root development. Am J Bot 99: 101-107. doi:10.3732/ajb.1100136. PubMed: 22174335. 22174335

[44] Yedidia I, Benhamou N, Chet I (1999) Induction of defense responses in cucumber plants (*Cucumis* *sativus* L.) by the biocontrol agent *Trichoderma* *harzianum* . Appl Environ Microbiol 65: 1061-1070. PubMed: 10049864. 10049864 10.1128/aem.65.3.1061-1070.1999 PMC91145

[45] Mach RL, Strauss J, Zeilinger S, Schindler M, Kubicek CP (1996) Carbon catabolite repression of xylanase I (*xyn1*) gene expression in *Trichoderma* *reesei* . Mol Microbiol 21: 1273-1281. doi:10.1046/j.1365-2958.1996.00094.x. PubMed: 8898395. 8898395

[46] St Leger RJ, Cooper RM, Charnley AK (1986) Cuticle-degrading enzymes of entomopathogenic fungi: Regulation of production of chitinolytic enzymes. J Gen Microbiol 132: 1509-1517.

[47] Fernandez J, Wright JD, Hartline D, Quispe CF, Madayiputhiya N et al. (2012) Principles of Carbon Catabolite Repression in the Rice Blast Fungus: Tps1, Nmr1-3, and a MATE–Family Pump Regulate Glucose Metabolism during Infection. PLOS Genet 8: e1002673. 22570632 10.1371/journal.pgen.1002673 PMC3342947

[48] Altomare C, Norvell WA, Bjorkman T, Harman GE (1999) Solubilization of Phosphates and Micronutrients by the Plant-Growth-Promoting and Biocontrol Fungus *Trichoderma* *harzianum* Rifai 1295-22. Appl Environ Microbiol 65: 2926-2933. PubMed: 10388685. 10388685 10.1128/aem.65.7.2926-2933.1999 PMC91438

[49] Mastouri F, Björkman T, Harman GE (2010) Seed Treatment with *Trichoderma* *harzianum* Alleviates Biotic, Abiotic, and Physiological Stresses in Germinating Seeds and Seedlings. Phytopathology 100: 1213-1221. doi:10.1094/PHYTO-03-10-0091. PubMed: 20649416. 20649416

[50] Contreras-Cornejo HA, Macías-Rodríguez L, Cortés-Penagos C, López-Bucio J (2009) *Trichoderma* *virens*, a Plant Beneficial Fungus, Enhances Biomass Production and Promotes Lateral Root Growth through an Auxin-Dependent Mechanism in Arabidopsis. Plant Physiol 149: 1579-1592. doi:10.1104/pp.108.130369. PubMed: 19176721. 19176721 PMC2649400

[51] Chacón MR, Rodríguez-Galán O, Benítez T, Sousa S, Rey M et al. (2007) Microscopic and transcriptome analyses of early colonization of tomato roots by *Trichoderma* *harzianum* . Int Microbiol 10: 19-27. PubMed: 17407057. 17407057

[52] Fang W, St Leger RJ (2010) RNA binding proteins mediate the ability of a fungus to adapt to the cold. Environ Microbiol 12: 810-820. doi:10.1111/j.1462-2920.2009.02127.x. PubMed: 20050869. 20050869

[53] Mukherjee PK, Nautiyal CS, Mukhopadhyay AN (2008) Molecular Mechanisms of Biocontrol by *Trichoderma* spp. In: Nautiyal C Dion P Molecular Mechanisms of Plant and Microbe Coexistence. Berlin Heidelberg: Springer Verlag pp. 243-262.

