# Supplementary material for: Metarhizium robertsii Produces an Extracellular Invertase (MrINV) That Plays a Pivotal Role in Rhizospheric Interactions and Root Colonization
Source: PLoS One. 2013 Oct 21;8(10):e78118. doi: 10.1371/journal.pone.0078118 (PMC3804458; doi:10.1371/journal.pone.0078118)
Supplement: Figure S1 — Verification of MrInv disruption and complement in M. robertsii 2575. A, The schematic diagram of DNA crossover and integration in the genome of wild-type and mutant strains. B, wt, wild-type stain; ⊿MrInv, the mutant in which MrInv was replaced with the bar selective marker by homologous recombination; ⊿MrInv-rv, a transformant in which the ⊿MrInv complemented by the MrInv genomic fragment. (PDF) [file pone.0078118.s001.pdf]

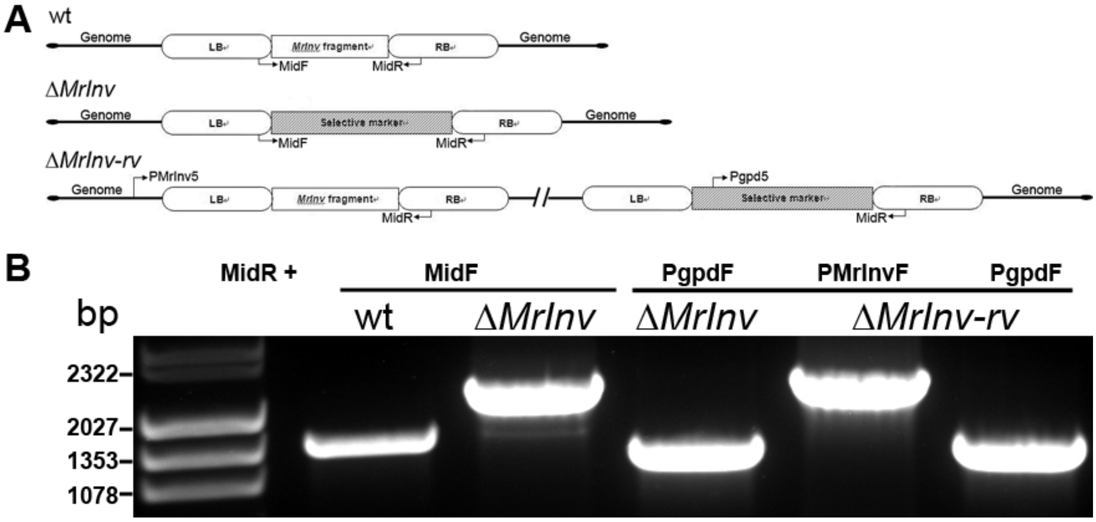

Figure S1 Verification of *MrInv* disruption and complement in *M. robertsii* 2575. A, The schematic diagram of DNA crossover and integration in the genome of wild-type and mutant strains. B, wt, wild-type stain; ⊿*MrInv*, the mutant in which *MrInv* was replaced with the *bar* selective marker by homologous recombination; ⊿*MrInv-rv*, a transformant in which the ⊿*MrInv* complemented by the *MrInv* genomic fragment.
