# Supplementary material for: Metarhizium robertsii Produces an Extracellular Invertase (MrINV) That Plays a Pivotal Role in Rhizospheric Interactions and Root Colonization
Source: PLoS One. 2013 Oct 21;8(10):e78118. doi: 10.1371/journal.pone.0078118 (PMC3804458; doi:10.1371/journal.pone.0078118)
Supplement: Figure S2 — Growth of M. robertsii 2575 wild-type strain (wt) and MrInv disruption mutant (⊿MrInv) on minimal medium agar plates supplemented with 1% sucrose at 27 °C for 10 d. ⊿MrInv grew comparatively less well than wild-type strain on sucrose. (PDF) [file pone.0078118.s002.pdf]

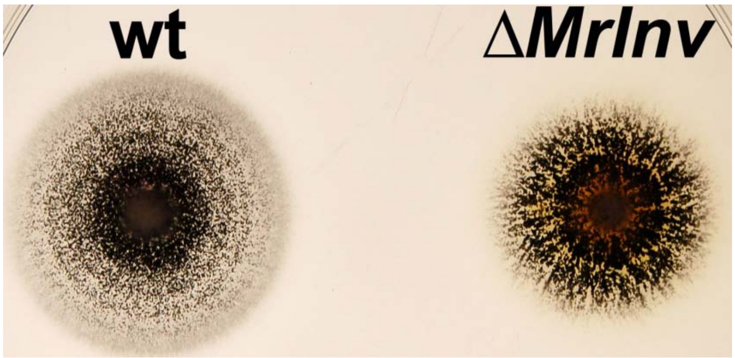

Figure S2 Growth of *M. robertsii* 2575 wild-type strain (wt) and *MrInv* disruption mutant (⊿*MrInv*) on minimal medium agar plates supplemented with 1% sucrose at 27 °C for 10 d. ⊿*MrInv* grew comparatively less well than wild-type strain on sucrose.
