# Supplementary material for: Metarhizium robertsii Produces an Extracellular Invertase (MrINV) That Plays a Pivotal Role in Rhizospheric Interactions and Root Colonization
Source: PLoS One. 2013 Oct 21;8(10):e78118. doi: 10.1371/journal.pone.0078118 (PMC3804458; doi:10.1371/journal.pone.0078118)
Supplement: Figure S4 — Switchgrass growth was monitored by measuring the shoot length and leaf chlorophyll content at one month intervals post-inoculation. Switchgrass were harvested after three months. The root length and plant dry biomass were determined. Nine plants from each pot were randomly selected for measurement. Values are means calculated from 27 replicates and bars represent the standard error. (PDF) [file pone.0078118.s004.pdf]

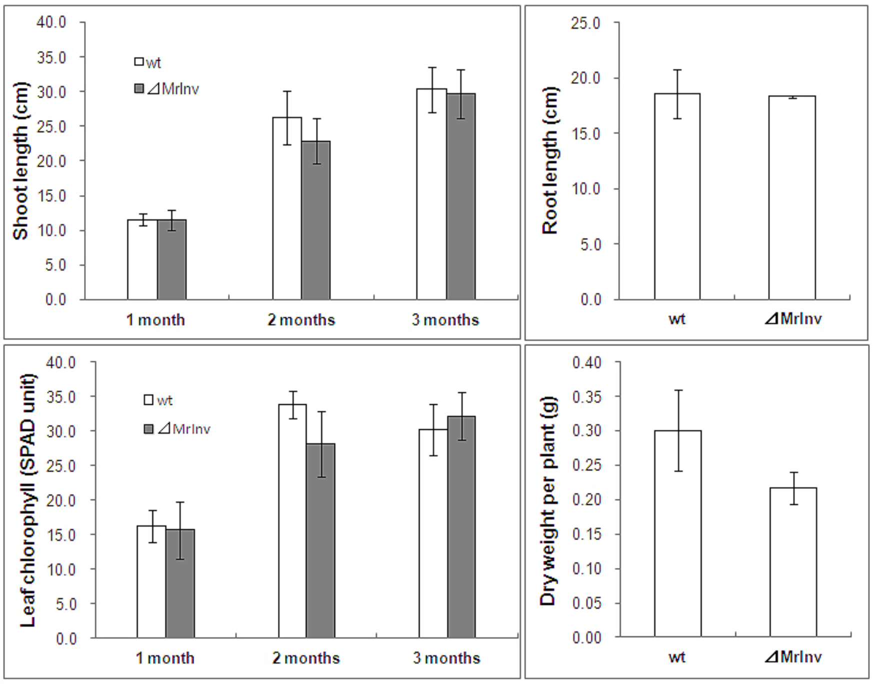

Figure S4 Switchgrass growth was monitored by measuring the shoot length and leaf chlorophyll content at one month intervals post-inoculation. Switchgrass were harvested after three months. The root length and plant dry biomass were determined. Nine plants from each pot were randomly selected for measurement. Values are means calculated from 27 replicates and bars represent the standard error.
