# Supplementary material for: Metarhizium robertsii Produces an Extracellular Invertase (MrINV) That Plays a Pivotal Role in Rhizospheric Interactions and Root Colonization
Source: PLoS One. 2013 Oct 21;8(10):e78118. doi: 10.1371/journal.pone.0078118 (PMC3804458; doi:10.1371/journal.pone.0078118)
Supplement: Table S1 — Primers used in this study. (PDF) [file pone.0078118.s005.pdf]

Table S1 Primers used in this study.

**Primers**	**Sequence**	**Application**	**Note**
MrInv_Lf	gcACTAGTttgagggcttccgtgacatg	The disruption of *MrInv*	*Spe* I
MrInv_Lr	gcACTAGTagttgctcggtatgacaggg		*Spe* I
MrInv_Rf	gcAGATCTcgtagtgcaatgagtctccc		*Bgl* II
MrInv_Rr	gcGATATCtttctggtagtctactgcgg		*Eco*R V
MrInv_C5	gcGATATCaggaggcatgctcggggtct	Plus MrInv_Rr to complement *ΔMrInv*	*Eco*R V
MidF	caaccaattgctctgttccc	Confirm the disruption of *MrInv* and	
MidR	cctctgtcaagatagaacgg	for the qPCR analysis of *MrInv*	
tps1f	tcaaggacgttcaagatggc	For the qPCR analysis of *tps*1	*
tps1r	tgcacagacggtttcttgag		
hxk1f	tcagtccaagtatcgcatgc	For the qPCR analysis of *hxk*1	*
hxk1r	agagccaacatcctccatgt		
gpd5	gactgcccgcattgagaag	House-keeping gene in *M. robertsii*	
gpd3	agatggaggagttggtgttg		
PMrInv5	tgggcactttgaggtatgcg	Confirm complement of *ΔMrInv*	
Pgpd5	ttgacaaggtcgttgcgt		

* Putative function based on sequence homology. Underlined letters indicate the restriction endonuclease cutting site.
